# What determines immigrant caregivers’ adherence to health recommendations from child primary care services? A grounded theory approach

**DOI:** 10.1017/S1463423619000033

**Published:** 2019-03-20

**Authors:** Susana Mourão, Sónia F. Bernardes

**Affiliations:** 1 Department of Social and Organizational Psychology, Instituto Universitário de Lisboa (ISCTE-IUL), Lisboa, Portugal; 2 Department of Social and Organizational Psychology, Centro de Investigação e Intervenção Social (CIS-IUL), Lisboa, Portugal

**Keywords:** child primary care, grounded theory, health recommendations, immigrants’, health, treatment adherence

## Abstract

**Aim:**

To investigate the diversity and specificity of the determinants of immigrant caregivers’ adherence to child primary care (CPC) health recommendations.

**Background:**

Immigrant caregiver’s adherence to CPC health recommendations is of utmost importance to minimize their children’s health-related vulnerabilities. Some research has been conducted on the determinants of immigrants’ access to health services, but much less is known about the determinants of their adherence to health professionals’ recommendations once they get there, especially in a primary health care context. This study contributes to bridge these gaps.

**Methods:**

Interviews and focus groups were conducted, with immigrant and non-immigrant caregivers living in Portugal (*n*=35), from heterogeneous socioeconomic backgrounds. Focus group and individual interview scripts were developed to explore caregivers’ understanding and use of CPC services and, particularly, their adherence to CPC recommendations. A socio-demographic questionnaire was also administered. Qualitative data were analyzed using a grounded theory methodology.

**Findings:**

‘Adherence to CPC health recommendations’ is a core and multidimensional concept. Several determinants were identified at individual, interpersonal, organizational and structural levels. Some determinants were highlighted both by immigrant and non-immigrant caregivers: valuing children’s health, usefulness of recommendations, perceived health-care professionals’ competence, central role of vaccination in CPC and caregivers’ socio-economic conditions. Other determinants were specifically mentioned by immigrant caregivers: expectations about traditional versus pharmacological treatments, cultural mismatches in children’s care practices, perceived quality of Portuguese CPC services versus CPC from countries of origin. These results provide innovative theoretical and empirical contributions to the field of primary health care and, particularly, to immigrant caregivers’ adherence behaviors. Implications for research on treatment adherence in primary care contexts, the development of interventions that promote caregivers’ adherence to CPC health recommendations and for child protection will be discussed.

## Introduction

Several studies have shown that immigrant children are at increased risk of developing health-related problems once they get into their host countries (eg, Mas *et al*., 2010; Schmeer, 2012). Child Primary Care services (CPC) play a fundamental role in the health promotion of such vulnerable children (Kuo *et al*., 2012). However, evidence suggests that immigrant children, along with their caregivers, often have trouble accessing or using primary care services (Sime, 2014; Ahmed *et al*., 2016) and also in adhering to CPC health recommendations (Gimeno-Feliu *et al*., 2009; Kirkpatrick *et al*., 2012; Heerman *et al*., 2016). Thus, it is of paramount importance to promote a better engagement of immigrant families with CPC and contribute to decrease their vulnerability regarding non-adherence to health recommendations.

Although much research has been conducted on the determinants of immigrants’ access to health services (Dias *et al*., 2008; 2018; Wafula and Snipes, 2014; Kalich *et al*., 2016; Oliveira and Gomes, 2018), there is much less research on the determinants of their adherence to health professionals’ recommendations once they get there, especially in a primary care context. Thus, the general goal of this study was to contribute to bridge these gaps by investigating the diversity and specificity of the determinants of immigrant caregivers’ adherence to CPC health recommendations.

### The relevance of adhering to CPC health recommendations

CPC has an essential role in promoting health equity by buffering the impact of (lower) socio-economic conditions on children’s health. This is so because most European CPC (Portuguese included) is universal and free to all children [Direção Geral da Saúde (DGS; National Institute of Health), 2009; van Esso *et al*., 2010] and plays a crucial role in the prevention and early detection of health-related problems with higher prevalence among immigrant and/or low-income children (eg, overweight/obesity, dental caries, mental health issues).

CPC are the primary technical source of knowledge and advice for parents (Barak *et al*., 2010). A preventive approach through anticipatory guidance is commonly incorporated into well-child visits; parents are informed of what to expect at the next stage of their child’s development and given recommendations about health issues (eg, nutrition, injury prevention, medication). These recommendations are often provided in conjunction with regular health screenings, immunization schedules, developmental surveillance and family psycho-social assessment. Thus, CPC recommendations assume in nowadays services a great relevance (DGS, 2013; Jenni, 2016; Garg *et al*., 2017) and children’s health might depend on the degree to which their families adhere to them, that is, their level of therapeutic adherence (Dunbar-Jacob *et al*., 2012; Straub, 2012). Consequently, promoting caregivers’ adherence to CPC recommendations is vital for the protection of (immigrant) children’s health.

Indeed, many studies show that poor therapeutic adherence is often associated with worse health, faster illness progression and recurrent hospitalizations and work/school absenteeism (Rodríguez-Gómez and Salas-Serrano, 2006; Dunbar-Jacob *et al*., 2012; Kuo *et al*., 2012). Thus, adhering to CPC recommendations is important not only to the general population, but particularly to immigrants and their children given their increased health-related vulnerabilities.

### Determinants of immigrant caregivers’ adherence to CPC health recommendations

To improve immigrant caregivers’ therapeutic adherence behavior a grasp of its main driving factors is needed. Mainstream research on treatment adherence, mostly drawing upon socio-cognitive models of health behavior change (eg, Conner and Norman, 2005), has focused on intentional non-adherence, that is the conscious decisions and/or lack of motivation to follow health professionals’ recommendations, and often disregarding the influence of social and contextual variables (Dunbar-Jacob *et al*., 2012; Brannon *et al*., 2014). Nevertheless, unintentional non-adherence, that is, non-adherence resulting from external and contextual constraints, may be particularly important to understand immigrants’ adherence behaviors (Martin *et al*., 2010).

Low socio-economic conditions, problems of communication and cultural differences have been consistently identified as important barriers to immigrants’ non-adherence, particularly when health systems are organized to serve the dominant culture (Straub, 2012; Mourão and Bernardes, 2014). Thus, ignoring the powerful influence of such contextual factors and attributing immigrants’ non-adherence exclusively to individual factors is, to a certain extent, ‘blaming the victim.’ As such, it is our contention that dominant socio-cognitive models on therapeutic adherence do not seem to be enough to account for immigrants’ treatment adherence behaviors, and an intensive analysis of the specific contextual factors associated to such pattern of behaviors is in need.

It should also be noted that, as most studies on treatment adherence have been focusing on non-immigrant adults’ pharmacological treatment adherence (eg, Colby *et al*., 2012; Robbins *et al*., 2012), very little is known on the determinants of immigrant (and non-immigrant) families’ adherence to CPC recommendations. Consequently, the first aim of this study was to investigate the diversity of determinants of immigrant caregivers’ adherence to CPC recommendations. We also aimed at undertaking a comparative analysis between the determinants of immigrant and non-immigrant caregivers’ adherence to CPC recommendations, as to be able to pinpoint some common and specific determinants of immigrant caregivers’ therapeutic adherence.

To achieve these goals we conducted a grounded theory study involving Cape Verdean and Brazilian immigrants in Portugal, as these are currently the most prominent immigrant groups in the country [Serviço de Estrangeiros e Fronteiras [(SEF) National Service from Foreign and Borders], 2016]. Like in other countries, if much research has been conducted on the determinants of immigrants’ access to Portuguese health services (Dias *et al*., 2008; 2018; Oliveira and Gomes, 2018), the evidence on what determines Cape Verdean and Brazilian caregivers’ adherence to CPC recommendation is, to the best of our knowledge, non-existent.

## Methods

### Data collection

Immigrant (Cape Verdean and Brazilian) and Portuguese caregivers participated in this study. All caregivers were recruited in the Lisbon Metropolitan Area that, according to the Portuguese Immigration and Borders Service, presents one of the highest rates of immigrants in the country (SEF, 2016).

Following Strauss and Corbin’s (1990) guidelines for grounded theory development, this study included two waves of data collection, so that the data collected and analyzed in the first wave could partially inform the second wave of data collection (eg, development of the interview script). In *the first wave*, five focus groups (FG) were conducted, with a maximum of six participants each (Krueger and Casey, 2000). Sessions were homogeneous in terms of participants’ nationalities; that is all Cape Verdean or all Brazilian. As it can be seen in Table 1, the FG script included general stimuli questions to tap into immigrants’ experiences with CPC in Portugal and in their countries of origin.

**Table 1:** Semi-structured focus group and individual interview scripts

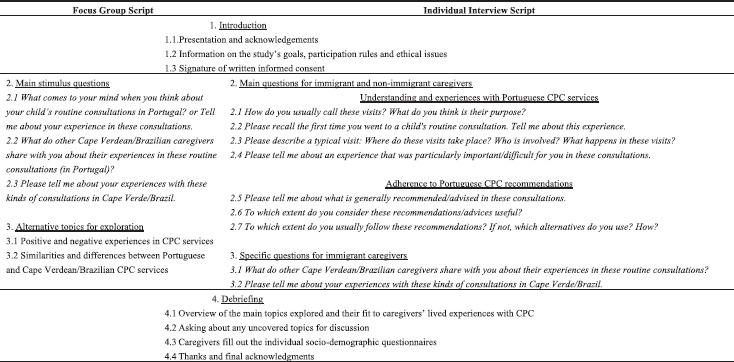

Note: CPC=child primary care.

Theoretical sampling (Strauss and Corbin, 1990) was used for the *second wave of data collection*, with the following inclusion criteria: immigrant caregivers from Cape Verde or Brazil; with children aged between three months and five years. In addition, caregivers’ socio-economic conditions (eg, years of education, profession, household income) and their use of public/private health systems was considered to ensure a more heterogeneous pool of participants. Portuguese caregivers, with similar socio-economic status (SES), were also included in this wave of data collection as to allow us to identify, among the determinants of adherence to CPC health recommendations, the ones that were shared by immigrant and non-immigrant caregivers and the ones that were exclusively mentioned by immigrant caregivers.

In total, 17 semi-structured individual interviews were conducted. A semi-structured interview script was developed to allow an in depth analysis of themes/categories that had previously emerged in the FG (Strauss and Corbin, 1990). The structure of this script was based on similar studies focusing on immigrants’ health-related issues (eg, Kong and Hsieh. 2012) and on the basic standards for interview script construction (eg, Creswell, 2007). A detailed description of the individual interview script is presented in Table 1.

All FG and interviews were recorded in audio format, and subsequently verbatim transcribed. The data collection stopped when saturation of information was reached.

After each FG and individual interview, participants were asked to individually fill out a brief questionnaire that collected their socio-demographic information (eg, sex, age, marital status, years of education) and, in the case of immigrants, information on their time and legal status in Portugal (please see questionnaire in Appendix).

### Data analysis

Qualitative data analysis was based on a grounded theory methodology, namely, on three different coding procedures: open, axial and selective (Strauss and Corbin, 1990). All qualitative data collected in *waves* 1 and 2 was analyzed following the same methodological procedures. Indeed, data collection and data analysis were an iterative process, but for the sake of clarity they are sequentially described below.

We started with a descriptive open coding: analyzing phrase by phrase and categorizing each unit of meaning as near as possible to participants’ speeches. Afterwards, we proceeded to a more conceptual open coding: categories representing a similar phenomenon were aggregated in more encompassing/abstract categories. For each conceptual category (eg, infant feeding), properties (eg, introduction of food in children’s diet) and their respective dimensions (eg, gradual versus random) were identified, whenever possible.

Axial coding was followed, that is to recognize interrelations between categories/concepts in participants’ speeches. Whenever possible, Strauss and Corbins’ coding paradigm was used for the identification of the most important axial categories. Each axial category represents a specific type of relationship with the principal phenomenon, in this case, adherence to CPC recommendations. Applying the coding paradigm to linguistic peculiarities in the data (eg, ‘because,’ ‘since,’ ‘as result of’) the following main axial categories were identified: (1) causal conditions, which contribute to the occurrence or development of the principal phenomenon. These causal conditions were coded as ‘determinants of adherence’ and varied in the extent to which their influence on adherence was direct (proximal determinants) or via other concepts (distal determinants); and (2) intervening conditions, which represent the social or cultural environment where the principal phenomenon occurs (eg, caregivers’ cultural background or socio-economic conditions).

**Figure 1:**
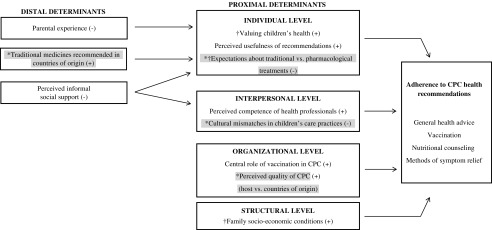
A grounded theory on the determinants of (immigrant) caregivers’ adherence to child primary care (CPC) health recommendations. **Note**: (+)=Positive association; (−)=Negative association; ∗= Specific to immigrants; †= Specific to lower socio-economic status.

Finally, a selective coding process took place and an integrated/overarching model on the determinants of caregivers’ adherence to CPC recommendations was developed, which is depicted in Figure 1. All data analysis was performed using the Atlas.Ti 6.2 software.

#### Quality criteria

Several criteria were used to ensure the quality of the qualitative data analysis and its results (Strauss and Corbin, 1990; Charmaz, 2006):

- Adequacy of references – all FG and interviews were audio taped and verbatim transcribed.
- Triangulation of participants – who had heterogeneous characteristics and diverse experiences in CPC.
- Triangulation of researchers – a second researcher was present in the FG to register important information about participants and to promote reflexivity. Additionally, the second author regularly analyzed the results and discrepancies in the interpretation of data were resolved by consensus.
- Developing memos and diagrams – several conceptual dated memos were created, explaining decisions about the development of categories over time. Operational memos were also produced, indicating what needed to be further explored in the following data collection moments. Finally, visual diagrams were developed, representing the associations between concepts.
- Peer and external consultation – two independent researchers analyzed the final definitions of categories, diagrams and associated speeches. One had previous experience in grounded theory analysis and the other in ‘cultural issues in health.’ Lastly, the process of analysis and preliminary results were presented/discussed in two research groups (one of them dedicated to grounded theory analysis).

Finally, it should be noted that descriptive and bivariate statistical analyses (*t*-test, One-way analysis of variance, Fisher’s Exact Test) were conducted to describe the sample of participants and to compare the socio-demographic characteristics of Portuguese, Cape Verdean and Brazilian Caregivers. These analyses were conducted with IBM-SPSS Statistics 24.0.

### Procedure

This study was approved by the Institutional Review Board of ISCTE – University Institute of Lisbon. The recruitment process started by contacting key-institutions in the Lisbon metropolitan area (eg, immigrant associations, National Center for Immigrant Support) as to identify eligible participants (Yin, 2011). Caregivers (eg, mothers, fathers, grandparents, legal guardians) who generally took the responsibility of taking their child/children to CPC services were requested to collaborate on a study about their experiences with child well-visits. Those who accepted to participate received a formal invitation one week before their FG/interview (Morgan and Krueger, 1998). At the end of each FG/interview, a ‘snowball’ strategy was also used (Yin, 2011). FG/interviews were conducted in neutral and comfortable spaces (eg, rooms in immigrant associations, participants’ households; Krueger and Casey, 2000).

As presented in Table 1, both scripts started by providing information on the study’s goals and participation rules (eg, freedom to stop participation at any time, without any personal implications). All participants signed a written consent, where the confidentiality and anonymity of data was guaranteed and they were informed about their voluntary participation. After the FG/interviews, participants individually filled out the socio-demographic questionnaire (in Appendix).

## Results

### Participants

In the *first wave of data collection*, 13 Cape Verdean (68.4%) and six Brazilian immigrant mothers (31.6%) participated in the study (*n*=19). Participants’ ages ranged between 22 and 41 years old (*M*=28.7; SD=6.2), they had arrived Portugal on average 7.3 years ago (SD=4.8) and most of them (73.7%) had kept their original nationality. They had between 1 and 7 children, with diverse ages. On average, they had a basic school level (*M*= 9.8 years; SD=3.3), the majority were employed (66.7%) as house/cleaning maids (55.6%) and half of them had a monthly household income of 485€ or less. The majority (94.1%) used the public health system and only a Brazilian mother reported using private services. As compared to Cape Verdeans, Brazilian mothers reported higher socio-economic conditions: more years of education, *t* (15.796)=−3.503, *P*=0.003; higher monthly household income, *P*=0.002, Fisher’s Exact Test; and better employment status (all Brazilian caregivers were employed while half of the Cape Verdean caregivers were unemployed), *P*=0.054, Fisher’s Exact Test.

In total, 16 mothers participated in the *second wave of data collection*: four Cape Verdean, seven Brazilian and five Portuguese; whose ages ranged between 18 and 48 years old (*M*=30.3; SD=7.3). On average, they had around 12 years of education (*M*=11.7 years; SD=4.0) and half of them were employed. They had heterogeneous professions (eg, cleaning/housemaid, store clerk, teacher) and their monthly household incomes were diverse ranging from less than 485€ (37.5%) to more than 1500€ (12.5%). Immigrants had arrived Portugal on average 10.7 years ago (SD=4.8) and half of them had national/double citizenship. One Portuguese grandmother was also interviewed, complementing the information obtained from the mother. Again, the majority of caregivers (68.8%) used the public health system, although four immigrant mothers (three of them Brazilian) and one Portuguese reported using private health services. Cape Verdean, Brazilian and Portuguese mothers shared similar socio-economic conditions, particularly in what concerns their years of education, *F*(2, 13)=0.630, *P*=0.548, and employment status, *P*=0.742, Fisher’s Exact Test.

It should be noted that participants with secondary or higher education degrees, employed, with intermediary or specialized professions (eg, hairdresser, sociologist) and higher household incomes were included in the higher SES category. All the remaining were included in the lower SES category.

## What determines (immigrant) caregivers’ adherence to CPC health recommendations?

The core concept of ‘Adherence to CPC Recommendations’ is complex and multidimensional (see Figure 1). It includes reported adherence to general health advices (ie, non-specified), but also related with specific recommendations: vaccination, nutritional counseling and methods of symptom relief.

The main determinants of adherence to CPC recommendations are represented in Figure 1. Determinants were categorized as proximal or distal. Proximal determinants refer to factors that are more directly associated (positively or negatively) with caregivers’ adherence. Distal determinants include factors that, by being positively or negatively associated with the proximal determinants, may be indirectly related with adherence.

Proximal determinants were also categorized at different levels of analysis: individual, interpersonal, organizational and structural. Individual determinants refer to caregivers’ expectations or beliefs about their child’s health or CPC recommendations. Interpersonal determinants refer to caregivers’ perceptions of their relationship with health-care professionals. Organizational determinants refer to caregivers’ perceptions of the organization/structure of the CPC services (eg, quality of assistance). Finally, structural determinants relate to caregivers’ socio-economic conditions.

Some of these determinants were reported both by immigrants and non-immigrants (henceforth, shared determinants), but immigrants specifically highlighted others (henceforth, specific determinants; gray shaded in Figure 1). We will begin by describing the shared determinants and afterwards the specific determinants.

### Shared determinants of caregivers’ adherence to CPC recommendations

#### Individual determinants: valuing children’s health and perceived usefulness of recommendations

Caregivers, particularly from lower SES, reported adhering to CPC recommendations because they value their children’s wellbeing and their healthy development, and also because they recognized the importance of CPC recommendations to their children’s health. This is mostly relevant regarding general health advices, vaccination or nutritional counseling.

The perceived usefulness of CPC recommendations was of utmost importance for first time mothers (versus mother with multiple children; distal determinant in Figure 1), because they were less experienced and reported more difficulties with children’s care. It was also associated with caregivers’ perceptions of informal social support in Portugal (also a distal determinant in Figure 1). Those who reported less instrumental or emotional support availability from family or friends, reported higher usefulness of nutritional counseling as it compensated for the absence of perceived support in child care:

*INT (Interviewer): And, usually, do these recommendations make sense to you? Do you usually follow them?*

*I3 (Interviewee 3): To me… Always! (…) immigrants, here, don’t have their family nearby, isn’t it? So, I don’t have my mother or my mother-in-law nearby, nobody… (…) I also don’t usually call to ask ‘What can I do now?’ So, something that is said… (…) that is shared (…) I try to follow it strictly (…)* (interview; Brazilian mother; lower SES; public health system)

#### Interpersonal determinants: perceived competence of health professionals

Caregivers’ perceptions of health professionals as competent, both technically (eg, diagnostic skills) and relationally (eg, availability or empathy), also influenced their adherence to CPC recommendations. This was particularly relevant to adherence to general health advices, nutritional counseling and to recommendations of methods of symptom relief.

Once more, caregivers’ perceptions of informal social support in Portugal played a role as a distal determinant of adherence to CPC recommendations (as presented in Figure 1). Those who perceived higher availability of support from family/friends reported lower perceived competence of health professionals:

*P2 (FG participant 2): (…) honestly, my son has a scheduled surgery (…) but I don’t want to do it here! (…) Especially with his family doctor because… I am the one who does the diagnosis (…) Our mothers, our grandmothers are kind of doctors… you know? (…) So…when my mum laid him down to change his diaper, he pressed his own belly and she said ‘He has a hernia’ (…) then I went with him several times to the doctor, he examined his belly (…) P3 (FG participant 3): Don’t you think that he should do an ultrasound or something like that? P2: (…) But here they don’t do that! He is just doing it, because (…) I asked…* (2^nd^ FG; Brazilian mothers; higher SES; public health system).

#### Organizational determinants: central role of vaccination in CPC

Several caregivers perceived the vaccination as a main function of CPC:

P5: I*n the routine consultations I always go to the vaccination room* (1st FG; Cape Verdean mothers; lower SES; public health system).

*I4: (…) he goes to the health care center to be vaccinated* (interview; Brazilian mother; lower SES; public health system).

This recognition was positively associated with their adherence to recommended vaccines; especially those that were paid by the national health system.

#### Structural determinants: family socio-economic conditions

Caregivers who reported lower socio-economic conditions also reported lower adherence to vaccination recommendations; especially the ones referring to vaccines not paid by the national health system, which needed to be bought by the caregivers. Caregivers also stressed the relation between their lower socio-economic conditions and their adherence to nutritional counseling:

*P6: I gave everything to my daughter (…) I don’t have money to buy those kind of things… It is expensive, expensive! (…) I have to give her rice, soup… Here, when she is one year and eight months you have to give them everything… I’m poor, I have to give her everything!* (1st FG; Cape Verdean mothers; lower SES; public health system).

Thus, caregivers’ perceived favorable socio-economic conditions of their families influenced their adherence to CPC recommendations, particularly to vaccination and nutritional counseling. Again, the reference to this determinant of adherence to CPC recommendations was particularly stressed by caregivers of lower SES.

### Specific determinants of immigrant caregivers’ adherence to CPC recommendations

#### Individual determinants: expectations about traditional versus pharmacological treatments

Cape Verdean caregivers of lower SES particularly believed that recommended pharmacological treatments to manage their children’s common symptoms (eg, cough) were more ineffective or prejudicial than traditional medicines from their countries of origin (ie, did not relieve symptoms appropriately or had habituation effects). When they expressed these expectations they reported lower adherence to recommended pharmacological methods and increased use of traditional medicines (eg, massage with olive oil to relieve baby cramps). However, they hid these practices from health professionals because they feared these would not be accepted:

*INT: Have you already told someone at your health care center about these methods? P1: No. INT: (…) Why not? (…) P5: For fear… (…) P2: They will not accept this… And they will scold us ‘(…) you shouldn’t do it (…) It could hurt the child…’ P4: They can even call the police… Especially now that it is possible to take children from their parents… (…) If you tell the doctor that your child has certain symptoms and that you made a home-medicine (…) s/he will write everything down, as a proof…* (1st FG; Cape Verdean mothers; lower SES; public health system).

Moreover, these traditional methods were considered as more effective and beneficial than the recommended pharmacological treatments particularly when lay people or health professionals from caregivers’ country of origin consistently recommended them (distal determinant in Figure 1):

*P1: My daughter had a cough… (…) So, I bought that medicine in the pharmacy and the cough didn’t stop. Then, an older woman (…) gave me a plant to make an infusion (…) I gave her the tea and three days after that her cough stopped (…) These are traditional medicines (…) that aren’t bad for our body… P2: We make our medicines, traditional from Cape Verde, and it works!* (1st FG; Cape Verdean mothers; lower SES; public health system).

#### Interpersonal determinants: cultural mismatches in children’s care practices

Besides the cultural differences in methods of symptom relief, cultural differences regarding infant feeding practices between Portugal and immigrants’ countries of origin were also reported. These cultural differences included mismatches in the order of introduction of food in children’s diet (gradual in Portugal versus random in countries of origin) and in the type of food fed to children. Immigrants who perceived these cultural mismatches reported lower adherence to nutritional counseling:

*P2: They [health professionals] talk about introducing soup, after a while the meat (…) In Brazil this doesn’t exist, right? Children eat soup when they are two months old… But this is also a cultural issue, because we don’t have the habit of eating soup before the main dish. (…) We eat rice, beans, meat, fish… P3: We also eat guava… P2: I didn’t make the soup as the doctor recommended; I made it by my own.* (2nd FG; Brazilian mothers; higher SES; private health service).

In these cases, instead of adhering to CPC nutritional counseling, immigrants adhered to what was recommended in their countries of origin and, once more, hid it from health professionals:

*P5: Cachupa [a traditional Cape Verdean stew], that is our food (…) with four months old. But for the doctor, only now she could eat it. P1: I eat it since I was a baby… P5: Something that they tell me that is only from one year old on… (…) they ask me ‘Is she eating it?’ I say ‘No.’ ‘Oh, very good! From now on she can eat it.’ I can’t say ‘Well, she is already eating it.’* (1st FG; Cape Verdean mothers; lower SES; public health system).

As regards cultural mismatches in methods of symptom relief, immigrants reported divergences between the pharmacological treatments recommended in Portugal and traditional medicines from Cape Verde (eg, tea). Similarly, immigrants who perceived these mismatches reported lower adherence to CPC recommendations. Instead, they adhered to what was recommended in their country of origin and hid it from health professionals.

#### Organizational determinants: perceived quality of services (host versus countries of origin)

Immigrants’ adherence to CPC recommendations was also associated with their perceptions of the quality of CPC in Portugal; namely in what concerned its structure, functioning and staff. Those who perceived better quality of Portuguese services, as compared to services of their countries of origin, reported higher adherence to general health advices and methods of symptom relief:

*P2: And another thing that I noticed is the (…) nurse’s availability, who followed my pregnancy and after. She gave me her personal telephone number, in case I needed something, I could call her… In Brazil, something like that doesn’t exist. When will a nurse give you her personal telephone number? Never. If you want to know something, you have to go to the hospital (…)* (3rd FG; Brazilian mothers; lower SES; public health system).

## Discussion

The aims of this study were to investigate the diversity of determinants of immigrant caregivers’ adherence to CPC health recommendations and to identify which of those determinants were specific to immigrant caregivers or shared by all caregivers (immigrants and non-immigrants). In line with the general concept of therapeutic adherence, our core concept – adherence to CPC health recommendations (Figure 1) – was complex and multidimensional (Dunbar-Jacob *et al*., 2012; Straub, 2012; Brannon *et al*., 2014). It included self-reported adherence to pharmacological recommendations (ie, vaccination and pharmacological treatments for symptom relief), but also recommendations regarding health behaviors and lifestyle practices (ie, general health advices, nutritional counseling and traditional methods of symptom relief). The identification of these specific recommendations speaks to their centrality for caregivers. Indeed, although other kinds of recommendations are provided by Portuguese CPC (DGS, 2013; eg, prevention of childhood accidents), these were not mentioned by the participants. This may indicate that they either did not recognize them as relevant or that health professionals are not explicitly and consistently recommending them.

Our findings also suggest that caregivers’ adherence is determined by multiple factors. These determinants may have a more direct or indirect influence (proximal versus distal determinants). They may also be facilitators or barriers to adherence behaviors. Such determinants were also identified at different levels of analysis: individual, interpersonal, organizational and structural, which suggests that to fully understand immigrant caregivers’ adherence behaviors it is paramount to consider the mutual relation between individual and contextual factors.

### Individual determinants

At an individual level, some determinants were identified as shared facilitators of caregivers’ adherence (valuing children’s health, perceived usefulness of CPC recommendations) and others as specific barriers to immigrant caregivers’ adherence (expectations of traditional versus pharmacological methods). Overall, these facilitators and barriers reflect what is commonly referred to in many socio-cognitive models as outcome expectancies (eg, Health Belief Model, Self-Efficacy Theory, Theory of Planned Behavior; for a review see Brannon *et al*., 2014), that is, individuals’ beliefs regarding the extent to which their adherence behaviors will or will not produce valuable outcomes. Also, the lack of previous parental experience was identified as a distal and shared facilitator. First-time mothers reported lower self-efficacy regarding children’s care practices and, consequently, reported higher adherence to CPC recommendations.

In sum, in line with the underpinnings of many socio-cognitive models of health behavior change, perceived self-efficacy, along with outcome expectancies, are main predictors of intentional, deliberate and purposeful actions (Martin *et al*., 2010; Dunbar-Jacob *et al*., 2012). However, our data clearly shows that several interpersonal, organizational, cultural and societal determinants, which go beyond the individual’s control, must also be considered to account for caregivers’ adherence behaviors.

### Interpersonal determinants

At the interpersonal level, health professionals’ technical and relational competence constituted a shared adherence facilitator. This is consistent with findings from recent reviews and meta-analysis showing that trust in health professionals and satisfaction with technical and/or relational care play an important role in accounting for individuals’ treatment adherence in general (Zolnierek and DiMatteo, 2009; Jack *et al*., 2010; Hall and Roter, 2011; Hillen *et al*., 2011; Sendt *et al*., 2015; O’ Rourke and O’ Brien, 2017).

Our findings also show, however, that a relationship characterized by cultural mismatches in child care practices is a specific barrier to immigrant caregivers’ adherence. More specifically, immigrant caregivers reported divergences between some biomedical practices (eg, pharmacological methods of symptom relief and rigid nutritional recommendations) and more traditional, folk practices; the latter being perceived as more effective and less harmful than the former. Again, our findings are in line with previous research showing that immigrant mothers tend to use and believe in the effectiveness of alternative health practices in infant care (eg, herbal remedies) that have been passed from generation to generation and are deeply embedded in their culture (Hannan, 2015). When such practices and beliefs are perceived as being in conflict with health professionals’ recommendations, however, may negatively influence the delivery of care and adherence behaviors. More specifically, these perceived mismatches along with high levels of mistrust in health professionals’ ability to accept and accommodate cultural specificities may be leading immigrant caregivers to hide their folk practices, while at the same time not adhering to CPC recommendations.

These findings are not surprising as physicians’ clinical encounters with ethnic minority patients are also often influenced by racial bias, stereotyping or uncertainty (Hall *et al*., 2015; Drewniak *et al*., 2017). This is particularly striking in clinical encounters involving immigrants with lower levels of integration, because they feel more discrimination and prejudice compared with their more integrated counterparts (Landrine and Klonoff, 2001; Zagefka *et al*., 2014). These perceptions of discrimination generate high levels of mistrust of the medical system, undermining immigrants’ adherence to physicians’ recommendations (Landrine and Klonoff, 2001; Dovidio *et al*., 2017; McQuaid and Landier, 2017). Such perceptions are also serious barriers to cultural sensitivity in clinical encounters. Health professionals’ non-judgmental approach toward immigrants’ alternative health practices is essential to an open and effective communication, which is crucial to safer and more informed clinical decisions regarding children’s care (Hannan, 2015).

Still at an interpersonal level, our findings showed that relationships with significant others can also play a (distal) role on caregivers’ adherence to CPC recommendations. Indeed, social support has been consistently associated with higher adherence to preventive behaviors and medical regimens, contributing to better health outcomes (Taylor, 2011; Sendt *et al*., 2015). Conversely, our findings suggest that perceived social support is a shared barrier to caregivers’ adherence to CPC health recommendations, especially when significant others’ health advices contradict health professionals’ recommendations by proposing alternative care practices (eg, folk medicines). Although our findings seem to be at odds with mainstream research on social support and treatment adherence, recent studies have indeed suggested that belonging or not to an ethnic minority group may moderate the association between social support and treatment adherence (Magrin *et al*., 2015). More specifically, among immigrants, higher perceived social support from significant others may bear a negative influence on treatment adherence, by being less aligned with health professionals’ recommendations.

### Organizational determinants

At an organizational level, the fact that vaccination was perceived as CPC services main function was a shared facilitator of caregivers’ adherence. This generalized perception can be playing an important part in achieving good Portuguese vaccination rates, which contributed to eradicate some preventable diseases (DGS, 2015). Nevertheless, this perception is at odds with information included in Portuguese CPC technical documents (DGS, 2013), which depict vaccination as one among several other activities (eg, promotion of a healthy psychomotor development). This again may suggest that health professionals are not emphasizing enough other relevant primary care practices and/or that they were not recognized as relevant by caregivers. Thus, regardless of the role of health professionals in raising awareness of CPC services and activities, it is also relevant to reflect on the role of caregivers’ representations of CPC services/activities on their adherence to its recommendations. Considering immigrant families, the construction of these representations may not be dissociated from their previous experiences with the health services of their countries of origin (Almeida *et al*., 2014; Garg *et al*., 2017). Indeed, immigrants’ lower familiarity with the health system in their host countries, which contributes to their lower access to health care (Terraza-Núñez *et al*., 2010; Cheng *et al*., 2015), may also influence their adherence behaviors (Llop-Gironés *et al*., 2014).

### Structural determinants

At a structural level, caregivers’ low SES was identified as a shared barrier of adherence to CPC health recommendations. This finding is in line with previous studies on adherence to medical treatments or life style changes (Colby *et al*., 2012; Picorelli *et al*., 2014; Sendt *et al*., 2015). Thus, although many individuals from minority ethnic groups most often present lower socio-economic conditions (Morrison and Bennet, 2009; Straub, 2012), caregivers’ SES is a determinant of adherence that may go over and beyond the influence of immigrant status.

### Limitations and implications for future research

Some limitations should be pointed out to this study. Despite all efforts to ensure the presence of a large number of participants in the FG (Morgan and Krueger, 1998), two FG were conducted with less than four participants, which may have curtailed the richness and diversity of the collected data. Also, all data were collected by the first author, who is a non-immigrant Portuguese and, hence, a member of the majority group. This may have increased the likelihood of immigrants’ discourses being influenced by social/cultural desirability. We tried to minimize this effect by collecting data in neutral spaces, which were not in any way related with Portuguese health services. Participants were also informed that the researchers played no direct role in the national health system. Nonetheless, future research may also involve cultural mediators or members from the immigrant communities in the data collection procedures.

Another limitation was related with the fact that most caregivers who accepted to participate in the study were mothers, despite this is not a specific inclusion criteria. Although this is not entirely surprising, as mothers are more often recognized as the main caregivers and users of services (eg, Andrade, 2008), ends up to limit our knowledge about the adherence behaviors of other types of caregivers, namely fathers/grandfathers. Thus, the inclusion of their perspectives should therefore be a topic of concern to further research. Finally, our proposed grounded theory is exclusively based on (immigrant) caregivers’ perspectives. Future studies focusing on CPC health-care professionals’ views are also needed to complement our findings.

Despite these limitations, this study has relevant theoretical and practical implications. To the best of our knowledge, this is one of the first studies identifying the determinants of (immigrant) caregivers’ adherence to CPC health recommendations in general, and in Portugal, in particular. Our proposed grounded theory on (immigrant) caregivers’ adherence to CPC highlights the need of integration between the classical socio-cognitive theories of health behavior (change) and theories that account for the impact of social and cultural dimensions on adherence behaviors. First, such integrative efforts may increase socio-cognitive models’ predictive ability, which are not always able to account for ethnic and socio-economic differences in adherence behaviors (Landrine and Klonoff, 2001; Brannon *et al*., 2014). Moreover, it stimulates reflections on the underexplored concept of non-intentional non-adherence (Martin *et al*., 2010; Dunbar-Jacob *et al*., 2012), which provides a better framework to understand immigrant caregivers’ experiences. Despite its contributions, our proposed integrative model will benefit from further empirical support, namely, the use of quantitative methodologies to disentangle shared and specific determinants with respect to the effects of SES and immigrant status on adherence to CPC health recommendations.

Our findings also bear implications to clinical practice, contributing to improve immigrant caregivers’ adherence to CPC health recommendations. Findings allow the identification of several (modifiable) contributing factors to non-adherence at different levels of analysis, which may help to develop interventions that, by taking in consideration the multiple determinants, may prove to be more effective. Our findings also show that although some factors may equally influence immigrant and non-immigrant caregivers’ adherence (eg, SES), some determinants are specific to particular cultural groups (eg, Cape Verdean’s ‘expectations about traditional versus pharmacological treatments’). This suggests that culturally sensitive approaches to the promotion of caregivers’ adherence to CPC recommendation are needed.

Finally, the identification of determinants specifically related to immigrant caregivers may also bear important practical implications to the child protection area. Indeed, our results show that cultural mismatches in child care practices may be concealed from health professionals, reducing caregivers’ adherence to recommendations, which ultimately may increase immigrant children’s health risks and likelihood of being undertreated. Increasing health professionals’ awareness to such cultural mismatches and their influence on immigrant caregivers’ adherence behaviors will contribute to prevent disparities in primary care, hence, decreasing immigrant children’s social/health vulnerabilities in line with the main mission of CPC (Kuo *et al*., 2012).

In conclusion, we believe that the present study constitutes an original theoretical and empirical contribution to treatment adherence literature by focusing on immigrant caregivers’ adherence behaviors in a primary health-care context. It clearly stresses the importance of considering unintentional non-adherence behaviors and calls for further research on the role of social and cultural determinants of adherence. Finally, it contributes to the promotion of immigrant caregivers’ adherence to CPC health recommendations and, consequently, to the protection of more vulnerable children.
